# Molecular identification and morphological variations of *Amblyomma lepidum* imported to Egypt, with notes about its potential distribution under climate change

**DOI:** 10.1007/s00436-024-08284-0

**Published:** 2024-07-17

**Authors:** Eman M. Abouelhassan, Sohair GadAllah, Marwa S. Kamel, Mahmoud Kamal, Hazem H. Elsayed, Nahla H. Sallam, Mohammed Okely

**Affiliations:** 1 Department of Parasitology, Faculty of Veterinary Medicine, Suez Canal University, Ismailia, 41522 Egypt; 2 Entomology Department, Faculty of Science, Ain Shams University, Abbassia, Cairo, 11566 Egypt; 3 Department of Plant Protection, Faculty of Agriculture, Suez Canal University, Ismailia, 41522 Egypt; 4 Department of Microbiology, Faculty of Science, Ain Shams University, Cairo, 11566 Egypt

**Keywords:** *Amblyomma lepidum*, Dromedary camels, Morphometrics, Cluster, Niche modeling, Egypt

## Abstract

The tick *Amblyomma lepidum* is an ectoparasite of veterinary importance due to its role in transmitting livestock diseases in Africa, including heartwater. This study was conducted in 2023 to monitor *Amblyomma* spp. infestation in dromedary camels imported from Somalia, Ethiopia, and Sudan to Egypt. This study inspected 200 camels at the Giza governorate’s camel market that had been imported from Somalia, 200 from Ethiopia, and 200 from Sudan for tick infestation. Specimens were identified using morphological characteristics and phylogenetic analyses of the 12S and 16S rRNA genes. Clusters were calculated using an unweighted pair-group method with arithmetic averages (UPGMA) dendrogram to group the specimens according to their morphometric characteristics. The morphometric analysis compared the body shape of ticks collected from different countries by analyzing dorsal features. Principal component analysis (PCA) and canonical variate analysis (CVA) were performed to obtain body shape variation among specimens from different countries. Results indicated that camels were infested by 57 males *Amblyomma lepidum*, and no female specimens were observed; among these specimens, one may have a morphological abnormality. The results suggest that *A. lepidum* specimens collected from camels imported to Egypt from African countries exhibit locally adapted morphology with variations among specimens, particularly variations in body size. This adaptation suggests minimal potential for genetic divergence. Ecological niche modeling was used to predict the areas in Africa with suitable climates for *A. lepidum*. The study confirmed that East African countries might have the most favorable climatic conditions for *A. lepidum* to thrive. Interestingly, the amount of rain during the wettest quarter (Bio16) had the strongest influence on the tick’s potential distribution, with suitability decreasing sharply as rainfall increased. Future predictions indicate that the climatic habitat suitability for *A. lepidum* will decrease under changing climate conditions. However, historical, current, and future predictions indicate no suitable climatic habitats for *A. lepidum* in Egypt. These findings demand continuous surveillance of *A. lepidum* in camel populations and the development of targeted strategies to manage tick infestations and prevent the spread of heartwater disease.

**Supplementary Information:**

The online version contains supplementary material available at 10.1007/s00436-024-08284-0.

## Introduction

Ticks have been considered important medical and veterinary ectoparasites of livestock worldwide (Jongejan and Uilenberg 2004). The *Amblyomma* genus, which is represented by approximately 137 species and distributed in Neotropical, Afrotropical, and Australasian faunal regions (Guglielmone et al. 2015; Soares et al. 2023), has been widely associated with the spread of several pathogens such as *Rickettsia*, *Ehrlichia*, and *Theileria* (Eberhardt et al. 2020; Mnisi et al. 2022; Smit et al. 2023). In Africa, *Ehrlichia ruminantium*, the causative agent of fatal heartwater disease, is transmitted by several *Amblyomma* species (Faburay et al. 2008; Esemu et al. 2013; Getange et al. 2021). Among these species, *Amblyomma lepidum* transmits *E. ruminantium*, which causes heartwater disease in goats, cattle, and sheep (Walker et al. 2003).

*Amblyomma lepidum* is a three-host tick that is distributed in East Africa in more arid savannah countries, especially in central and eastern Sudan, Ethiopia, southern Somalia, eastern Uganda, Kenya, and the northern region of central Tanzania (Hoogstraal 1956; Walker et al. 2003). This species was introduced from Sudan and East Africa with imported cattle into Egypt but has not been established yet (Hoogstraal 1952; Liebish et al. 1989; Okely et al. 2022b). Recently, several studies collected *A. lepidum* from Egypt, but only male specimens were recorded (Youssef et al. 2015; Hassan et al. 2017; Okely et al. 2021; Abouelhassan et al. 2023).

Morphological and genetic variations within the same tick species from different geographic areas can occur due to adaptation to environmental conditions (Dantas-Torres et al. 2013). Variations in scutal ornamentation among *Amblyomma* species have been observed in African countries, such as *A. variegatum* and *A. tholloni* (Hoogstraal 1956). Morphometrics and morphological analyses have been conducted to examine shape variations in other *Amblyomma* species such as *A. mixtum*, *A. gemma*,* A. variegatum*, and* A. hebraeum* (Pretorius and Clarke 2001; Aguilar-Domínguez et al. 2021b). However, no study has yet investigated variations within the *A. lepidum* population.

The ecological niche modeling technique was used to understand the distribution pattern of disease vectors (Peterson et al. 2004). In recent years, several studies have anticipated the current and future potential distribution of tick vectors of medical and veterinary importance belonging to different genera (Boorgula et al. 2020; Aguilar-Domínguez et al. 2021a; Polo et al. 2021; Gillingham et al. 2023; Noll et al. 2023). However, no study has predicted the potential distribution of *A. lepidum* in its geographical range.

Here, we report the occurrence of *A. lepidum* in Egypt imported from three African countries (Ethiopia, Somalia, and Sudan) based on morphological and molecular characterization of ticks. We also describe shape variations in the imported ticks and employ climatic niche models to estimate historically suitable climatic habitats for *A. lepidum* across Africa. This approach allows us to identify areas where the vector may have thrived in the past and to assess potential shifts in its climatic suitability under current and future climate change scenarios. By evaluating the climatic suitability for *A. lepidum* in Egypt, we can determine whether the newly discovered samples are capable of establishing a population in Egyptian climates. This information is crucial for constructing proactive measures to manage the vector and mitigate its impact.

## Materials and methods

### Specimen collection and morphological identification

As a continuation of the collection trips to monitor *Amblyomma* ticks in Egypt (Abouelhassan et al. 2023), we examined dromedary camels monthly in 2023 in Giza governorate (Supplementary file 1), imported from three African countries: Somalia, Ethiopia, and Sudan. Six hundred (600) dromedary camels were inspected: 200 camels imported from Somalia, 200 from Ethiopia, and 200 from Sudan at the camel market in Giza governorate. All *Amblyomma* tick specimens were removed from camels using fine forceps and stored in vials containing 70% alcohol and 20% glycerol for transportation to the Okely’s Tick Collection (Department of Entomology, Ain Shams University, Cairo, Egypt) for morphological identification. Specimens were individually examined under a Labomed CZM4 Stereo Microscope (Labomed, Fremont, CA, USA) to identify them to species level based on morphological characteristics and taxonomic keys (Hoogstraal 1956; Walker et al. 2003; Okely et al. 2021; Abouelhassan et al. 2023).

### Imaging and morphological feature digitization

**Fig. 1:**
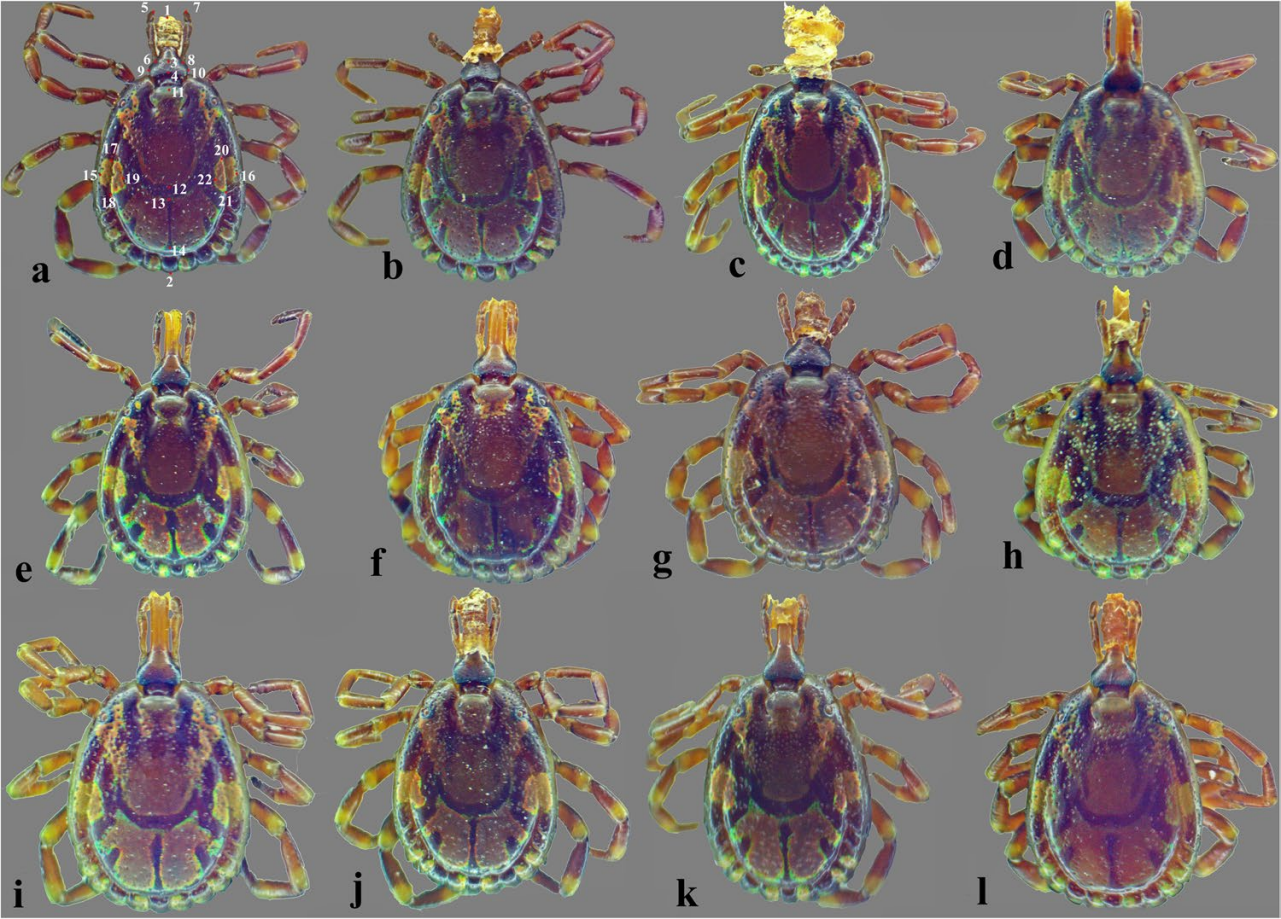
**a–l** Dorsal view of male *Amblyomma lepidum* ticks; specimen no. (a) with 22 morphological traits

**Fig. 2:**
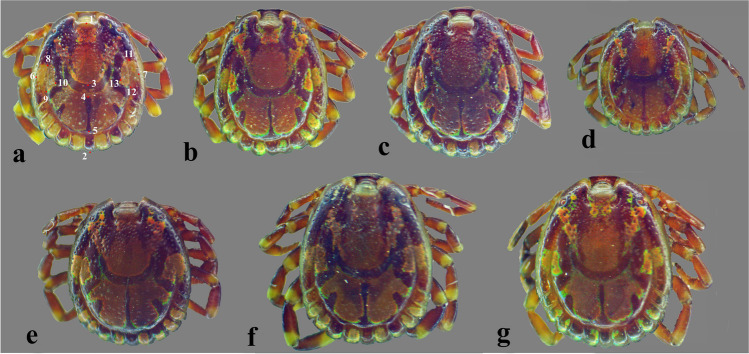
**a–g** Dorsal view of male *Amblyomma lepidum* ticks without mouthparts; specimen no. (a) with 13 morphological traits

An Am Scope MU1000 10MP Microscopic camera (Am Scope, Irvine, CA, USA) linked to a Labomed CZM4 Stereo Microscope (Labomed, Fremont, CA, USA) was used to photograph specimens at multiple focal planes. Selected morphological keys were manually digitized (Figs. 1a and 2a). Procrustes analysis of variance (ANOVA) in MorphoJ v.1.07 software (Klingenberg 2011) was conducted to reduce errors in morphological key imaging due to differences in scale, position, and orientation from key coordinates.

### Measurements and terminology

We measured linear distances on the image and clicked on any two keys to obtain any distance using tpsDig v2.16 software (Rohlf 2010). The measured characters were as follows: (1) body length (BL), (2) body width (BW), (3) palpi total length (TL), (4) length segment I (LSI), (5) length segment II (LSII), (6) length segment III (LSIII), (7) hypostome length (HL), (8) mesial area length (MAL), (9) lateral median area length (LMAL), (10) lateral median area width (LMAW), (11) posteromedian stripe length (POSL), (12) basis capituli ventral length (BCVL), (13) basis capituli ventral width (BCVW), (14) basic capituli dorsal length (BCDL), and (15) basic capituli dorsal width (BCDW). Fifteen morphological variables were measured for thirty *A. lepidum* male specimens. Abbreviations of the morphological characteristics follow previous literature (Walker et al. 2003; Aguilar-Domínguez et al. 2021b; Okely et al. 2021).

### Morphometric analysis

To evaluate shape variation among specimens from three different countries, PCA was performed to obtain shape changes; CVA was also conducted to assess the difference in body shape according to geographical variations. The tps files were transported to MorphoJ software (Klingenberg 2011). We used the Procrustes fit function and edited classifiers, and then PCA was used to observe the total shape variation in transformation grids. Boxplots were constructed to illustrate the variation in the size of body parts of specimens from each country.

### Phenetic relationships among specimens

To examine phenetic relationships among specimens based on morphological measurements, a UPGMA dendrogram was constructed based on different similarity indices by PCA using PAST software v 4.03 (Hammer et al. 2001).

### Genetic analysis

A total of 18 *A. lepidum* samples from the current study were analyzed. DNA was extracted from individual tick samples using the QIAamp DNA Mini Kit (Qiagen) according to the manufacturer’s instructions and then stored at − 20 °C until use. PCR was performed to amplify 16S rRNA and 12S rRNA genes. The primers used for the 16S rRNA gene were (5′-TTGGGCAAGAAGACCCTATGAA-3′ and 5′-CCGGTCTGAACTCAGATCAAGT-3), while those for the 12S rRNA gene were (5′-GAGGAATTTGCTCTGTAATGG-3′ and 5′-AAGAGTGACGGGCGATATGT-3′) according to Norris et al. (1999).

The amplified PCR products were checked using a 1.6% agarose gel containing 0.4 µg/ml of ethidium bromide. Sanger sequencing was accomplished by Solgent (Daejeon, South Korea). The generated sequences of 16S rRNA and 12S rRNA genes were analyzed using BLAST (Johnson et al. 2008). The nucleotide sequences were submitted to GenBank and then aligned and compared with closely related reference sequences retrieved from the GenBank database. The phylogenetic tree of 12S rRNA was constructed using a total of 42 sequences, including nine *Amblyomma lepidum* from the current study, 31 *Amblyomma* spp. from the GenBank database, and two *Ixodes* spp. sequences as an outgroup for rooting the phylogenetic tree. The phylogenetic tree of 16S rRNA was constructed using a total of 40 sequences, including nine *Amblyomma lepidum* from the current study, 29 *Amblyomma* spp. from the GenBank database, and two *Ixodes* spp. sequences as an outgroup for rooting the phylogenetic tree.

The phylogenetic trees were computed in IQTREE version 1.6.12 (Nguyen et al. 2015) using the maximum likelihood method, ModelFinder (Kalyaanamoorthy et al. 2017) to select the best model(s), and 2000 bootstrap replications. In this analysis, the best-fit model was K3Pu + F + G4, selected according to the Bayesian information criterion (BIC). The phylogenetic trees were visualized in MEGA11 software (Tamura et al. 2021).

### Ecological niche modeling

Occurrence records for *A. lepidum* were compiled from VectorMap (www.vectormap.org; 530 records), the Global Biodiversity Information Facility (GBIF; www.gbif.org; 7 records), and data from Egyptian tick surveillance programs conducted by Mohammed Okely (M.O.) during the years 2019 to 2023 and documented in previous literature (Okely et al. 2021; Abouelhassan et al. 2023), including one record from Egypt. These 538 records were merged and underwent rigorous cleaning to minimize biases and overpredictions in current and future model estimations (Okely et al. 2020). Only records with precise coordinates and metadata indicating their curation source were retained. Duplicates were removed using Microsoft Excel and spatially rarefied using SDMtoolbox 2.4 within ArcGIS 10.3 to eliminate redundant data occurring within ≤ 2.5′ (≈ 5 km^2^) (Brown et al. 2017; Okely and Al-Khalaf 2022). This yielded 517 unique, spatially rarefied records, randomly divided into calibration (259) and testing (258) subsets.

Environmental data for historical climatic conditions were sourced from WorldClim version 2.1 (www.worldclim.org) at a 2.5 min (≈5 km^2^) spatial resolution. Parallel data were obtained for two climate change scenarios: BCC-CSM2-MR (Beijing Climate Center Climate System) and IPSL-CM6A-LR (Institute Pierre-Simon Laplace Climate Model), representing climatic responses to ongoing climate change in the current period (2021–2041) and three future periods (2041–2060, 2061–2080, and 2081–2100). The two most pessimistic socioeconomic pathways (SSP.370 and SSP.585) were selected for these scenarios. Sixteen future scenario combinations (2 SSPs × 4 time periods × 2 scenarios) were utilized to describe current and future climatic conditions under changing climate. These datasets (historical and climate change scenarios) encompassed 19 bioclimatic variables derived from monthly temperature and precipitation records from 1970 to 2000 (Escobar et al. 2014) for the historical dataset. Parallel anticipations for these variables were available for the two climate change scenarios in each period and SSP. Notably, the Mean Temperature of the Wettest Quarter (Bio.8), Mean Temperature of the Driest Quarter (Bio.9), Precipitation of the Warmest Quarter (Bio.18), and Precipitation of the Coldest Quarter (Bio.19) were excluded due to spatial artifacts (Datta et al. 2020; Okely et al. 2023). Highly correlated variables were omitted using Pearson correlation (*r* >|0.7|; Dormann et al. 2013). The final variables for predicting *A. lepidum* climatic habitat suitability were Annual Mean Temperature (Bio.1), Isothermality (Bio.3), Temperature Seasonality (Bio.4), Min Temperature of Coldest Month (Bio.6), and Precipitation of the wettest quarter (Bio.16). Each bioclimatic layer was clipped to the study area (“Africa”) using the “extract by mask” tool implemented in ArcGIS 10.3 (Nasser et al. 2019) for historical and climate change scenarios. It is worth noting that the historical data (1970–2000) may not fully capture recent climate change trends. However, the model calibrated with this data is projected onto current (2021–2040) and future (2041–2100) under the Shared Socioeconomic Pathways (SSPs), representing anticipated climatic conditions under various climate change scenarios. A comprehensive list of the 19 bioclimatic variables is available in the supplementary file 2. The historical climatic niche model for *A. lepidum* was developed using the maximum entropy algorithm (MaxEnt v3.3.3e; Phillips et al. 2006), calibrating 259 occurrences with five bioclimatic variables (Bio1, 3, 4, 6, and 16) derived from the Pearson correlation coefficient thresholding and clipped to the African study area. The median across 100 bootstrap model replicates represented the species’ climatic habitat suitability under historical conditions. Model accuracy was evaluated using three approaches: the area under the curve (AUC; 0–1; Swets 1988; Nasser et al. 2021) where AUC = 0.5 indicates the model has no predictive ability and performs no better than random guessing; AUC = 1 represents a perfect model that can perfectly discriminate between positive and negative cases; and AUC > 0.5 shows the model has some predictive ability, with higher values indicating better performance. Partial receiver operating characteristic (*pROC*) statistics, with 500 bootstrapped iterations (Osorio-Olvera et al. 2018) in NicheToolbox, and the True Skill Statistic (TSS; -1 to 1; Allouche et al. 2006) were also used. Positive TSS values indicate strong agreement between predicted models and actual species distribution. To assess shifts in climatic habitat suitability due to current and future climate change, the historical MaxEnt model was projected onto the anticipated climatic conditions. Medians across the two models of each scenario at each period and SSPs were calculated using ArcGIS 10.3, mitigating the uncertainty of a single climate model (Shao et al. 2022). These medians represented the species’ climatic habitat suitability under climate change. Future models were estimated for 2041–2060, 2061–2080, and 2081–2100 for two SSPs (370 and 585), while 2021–2040 represented ongoing climate change conditions for the same SSPs.

## Results

**Fig. 3:**
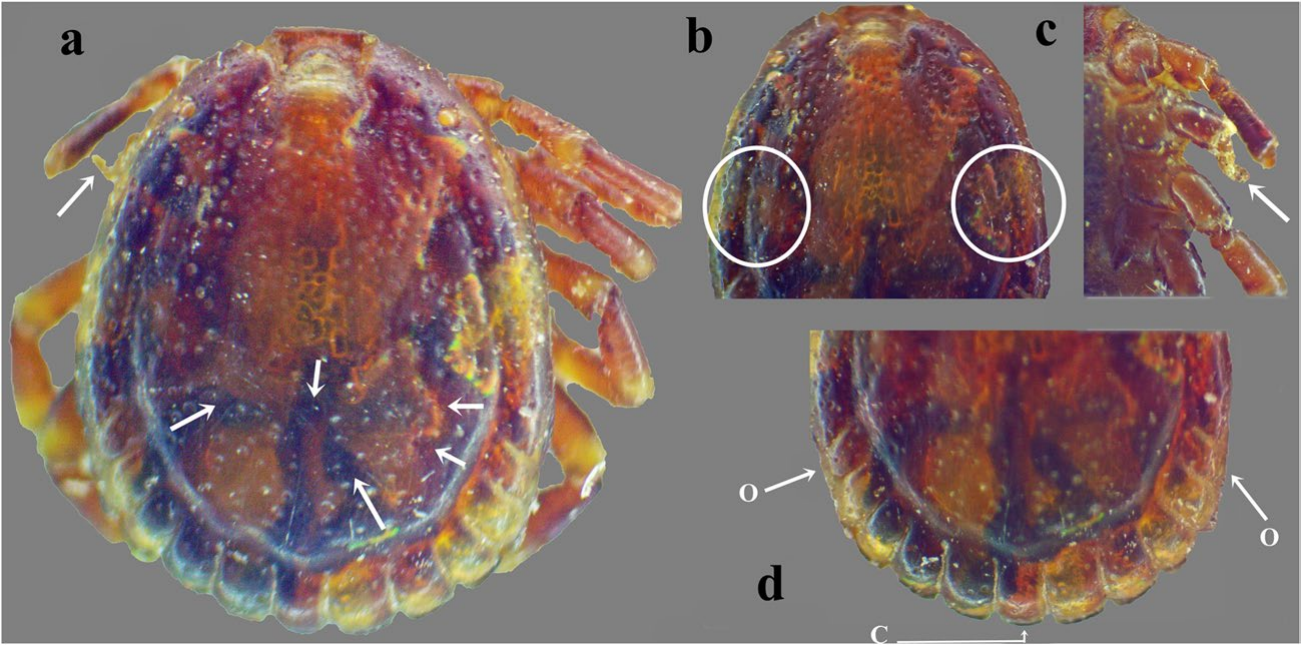
Abnormalities in male *Amblyomma lepidum* collected from imported camels to Egypt. **a** Abnormality in the enamel ornamentation on the conscutum with indistinct posteromedian stripe. **b** Indistinct enamel ornamentation of the lateral median area on the left side. **c** Atrophy of the second left leg. **d** Abnormality in festoon enameling

In total, 57 male *Amblyomma* ticks were recorded without female specimens being observed. All specimens were identified as *A. lepidum* based on the mesial area of enamel ornamentation with dense, coarse punctations, lateral median areas of enamel ornamentation, and festoons with enamel on 6–8 of 11 festoons (Figs. 1 and 2). Morphological variations in body shape among specimens were observed (Figs. 1 and 2). Interestingly, one specimen may have a morphological abnormality represented by an indistinct posteromedian stripe, atrophy of the second left leg, indistinct lateral median area of enamel ornamentation on the conscutum on the left side, and abnormality in the enamel ornamentation on the conscutum (Fig. 3a–c). There was also an abnormality in festoon enameling, where the central festoon and the outer festoon on the right side have ornamentation, although usually, there is no enamel on the central and two outermost festoons (Fig. 3d).

**Fig. 4:**
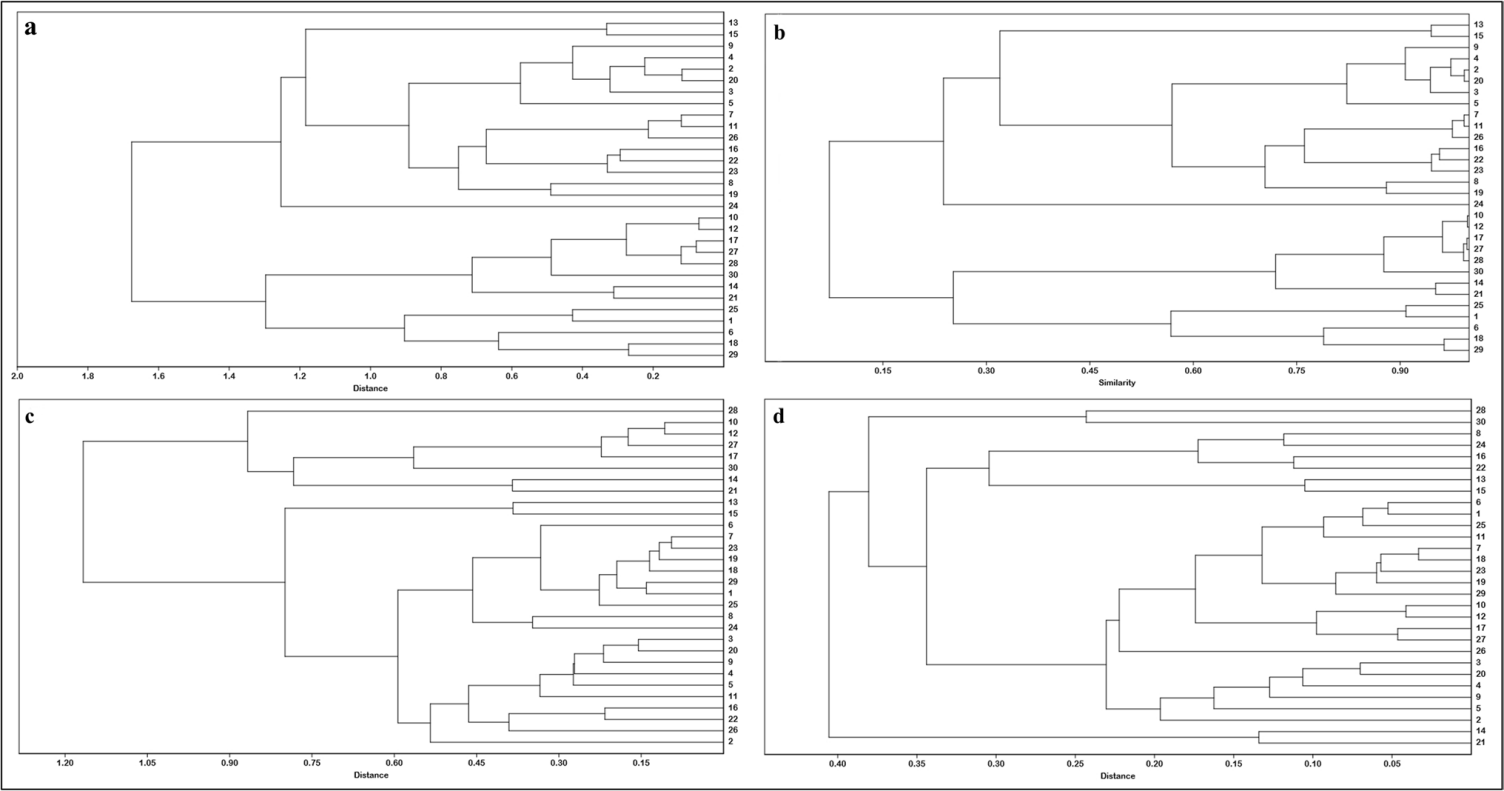
Cluster of the morphometry for *Amblyomma lepidum* males in four similarity indices

The measurements for all fifteen morphological variables for thirty *A. lepidum* (Supplementary file 3) were subjected to principal component analysis (PCA) due to correlations among some of these variables; the first 3 principal components (PCs) were used to estimate the cluster and correlation analyses. The first 3 PCs summarized about 90% of the overall variance in morphological data. Based on the morphometric measurements, the *A. lepidum* males were grouped into different groups with different similarity indices (Fig. 4). Pearson correlation coefficients between fifteen characters were calculated (Supplementary file 4). There is a highly negative correlation among different morphological traits and a positive correlation among other morphological traits. The highest positive correlation was detected between basis capituli ventral width and basic capituli dorsal width.

**Fig. 5:**
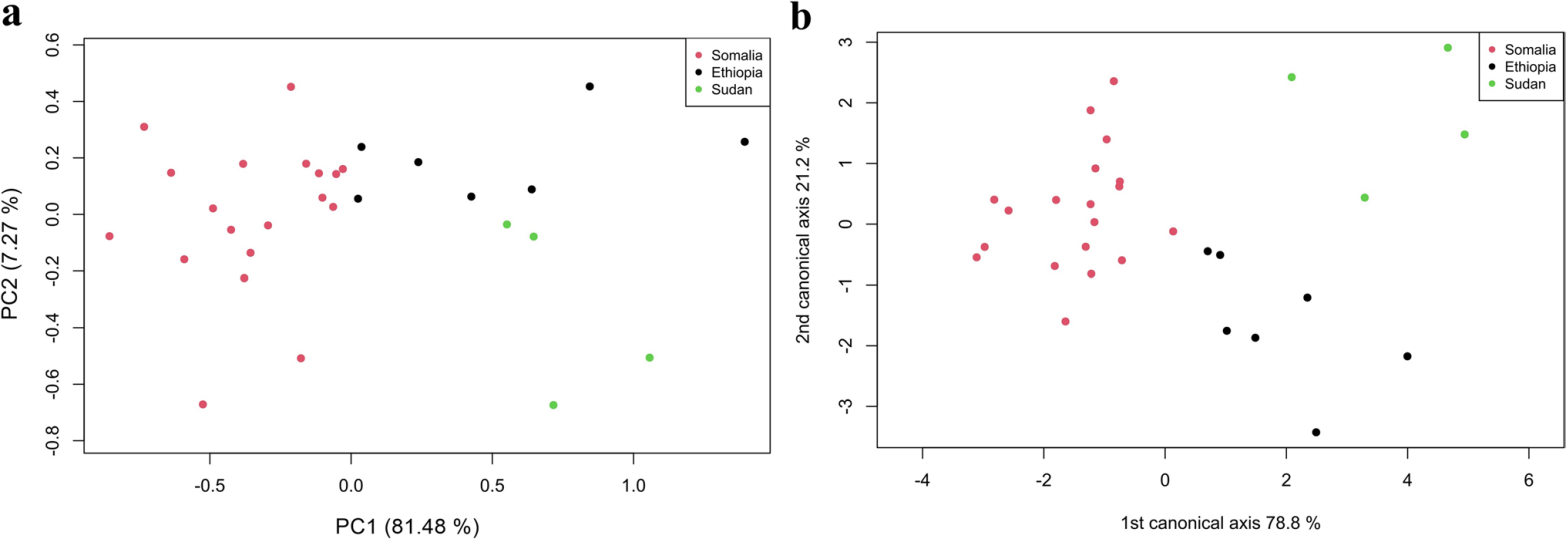
Analyses of the shape variation of dorsal views of male *Amblyomma lepidum* imported from three African countries to Egypt. **a** Principal component analysis (PCA). **b** Canonical variate analysis (CVA)

**Fig. 6:**
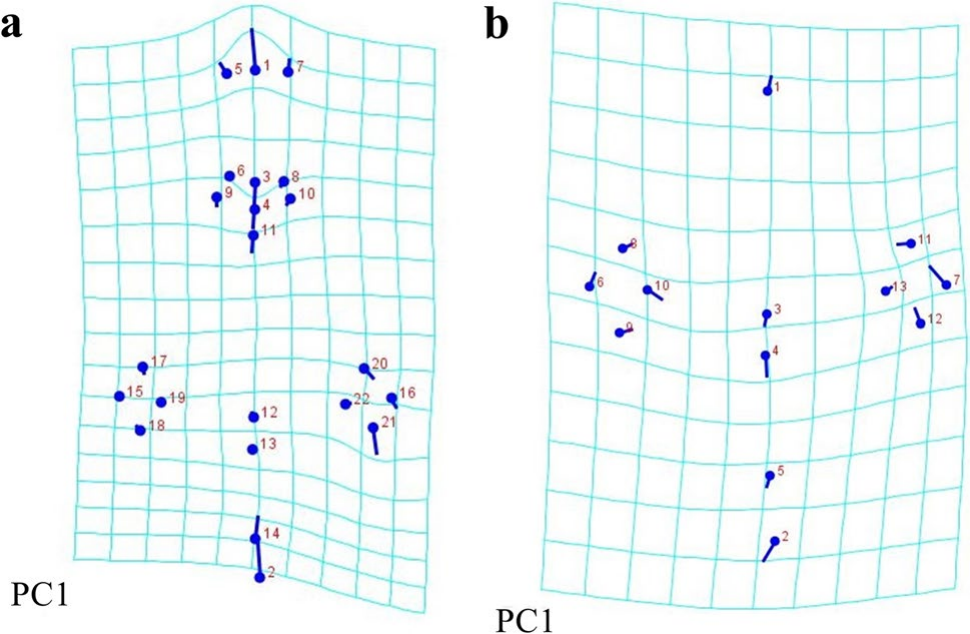
Transformation grids for visualizing a shape change for the first principal component. **a** Among *Amblyomma lepidum* males with mouthparts. **b** Among *Amblyomma lepidum* males without mouthparts

Principal component analysis (PCA) and canonical variate analysis (CVA) supported significant morphological differentiation (*P* < 0.05) between *A. lepidum* from Sudan, Somalia, and Ethiopia. The PCA showed shape variations, and the three groups separated distinctly, especially Somalia vs. Sudan/Ethiopia along PC1 (Fig. 5a). The CVA showed that the most significant variation was between Somalia vs. Sudan/Ethiopia along CV1 (Fig. 5b). The deformation grid of the first principal component for specimens with mouthparts revealed significant differences in feature numbers 1, 2, 14, 21, 3, 4, and 11 (Fig. 6a), whereas the deformation grid of the first principal component for specimens without mouthparts revealed significant differences in feature numbers 7, 2, 4, 10, and 12 (Fig. 6b).

**Fig. 7:**
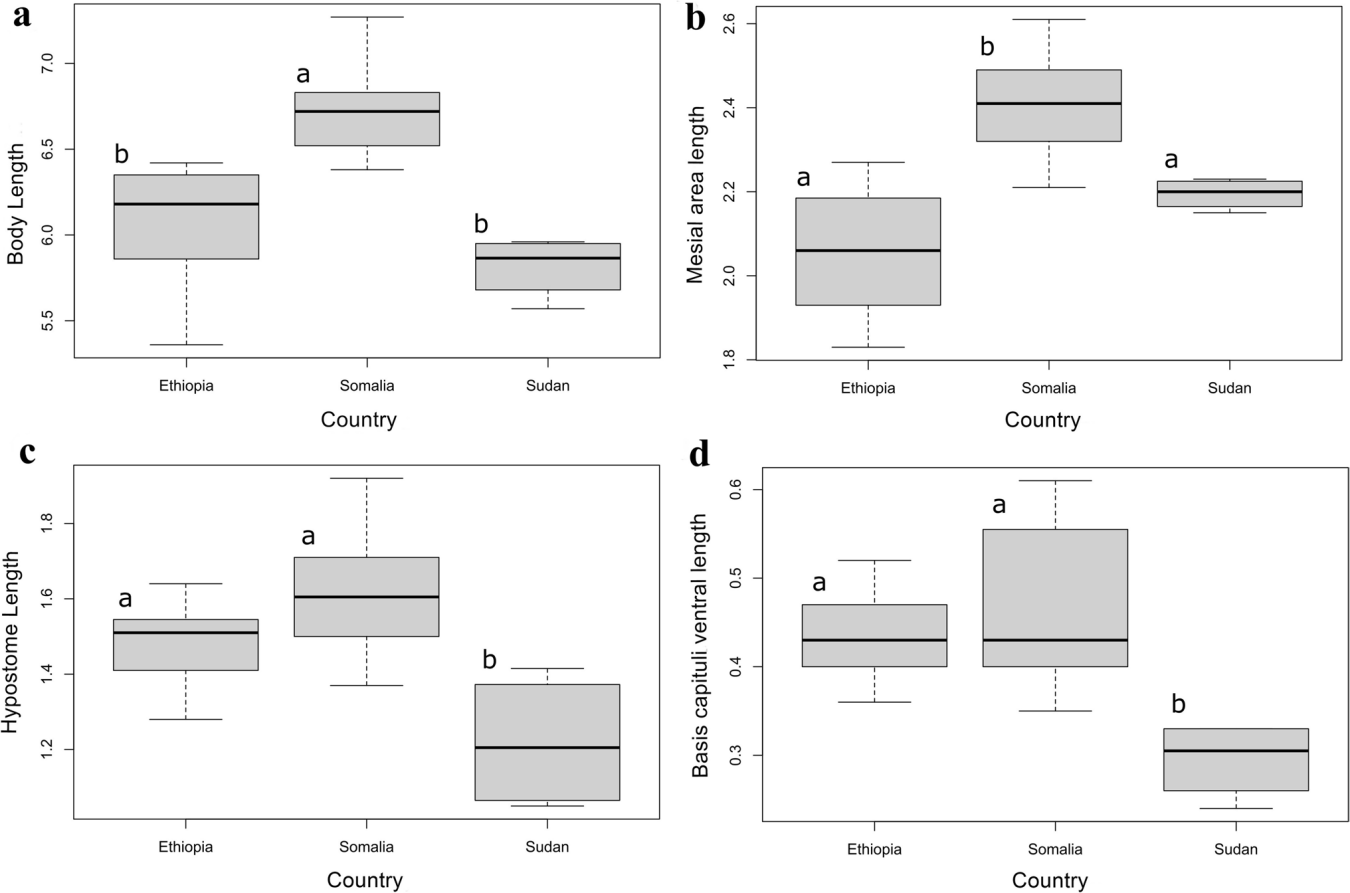
Box plots of variation in the size of body parts of *Amblyomma lepidum* male imported to Egypt from Sudan, Ethiopia, and Somalia. **a** Body length. **b** Mesial area length. **c** Hypostome length. **d** Basis capituli ventral length

There were significant differences between measurements for the morphological characters of *A. lepidum* ticks imported from the three countries (*P* < 0.05). The main differences among the fifteen morphological characters seem to be related to length measurements (body, capitulum, and hypostome), with Somalian ticks generally larger than Ethiopian and Sudanese ticks (Fig. 7).

The identification of tick species was confirmed through phylogenetic analysis of the 12S rRNA and 16S rRNA genes. The sequences for 12S rRNA and 16S rRNA were submitted to GenBank. Assigned accession numbers for 16S rRNA are OQ947777-85, and those for 12S rRNA are OQ955297-305. The lengths of amplified 12S rRNA and 16S rRNA sequences in all examined species in Egypt were similar (approx. 300 bp); the alignment length was ca. 250 bp after trimming the low-quality ends of each sequence.

**Fig. 8:**
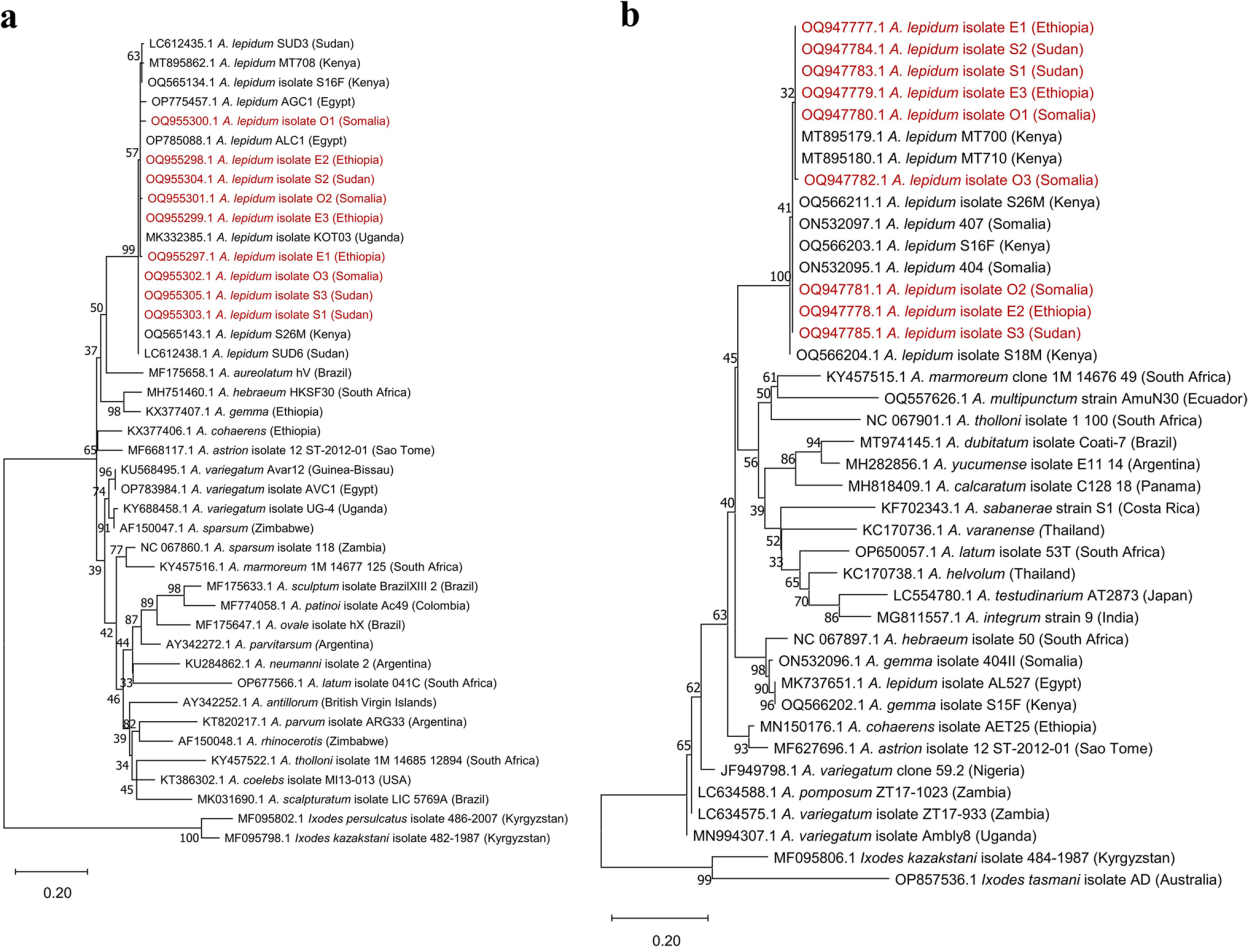
Maximum likelihood phylogenetic analysis of *Amblyomma lepidum* sequences imported to Egypt from Somalia, Ethiopia, and Sudan (OQ947777-85, OQ955297-305), and other tick species from GenBank. **a** Phylogenetic tree based on 12S rRNA sequences. **b** Phylogenetic tree based on 16S rRNA sequences

According to phylogenetic analysis, the 12S rRNA sequences of *A. lepidum* recorded in imported camels were closely related to specimens of *A. lepidum* from Uganda (MK332385), Egypt (OP785088 and OP775457), Sudan (LC612438 and LC612435), and Kenya (OQ565134, OQ565143, and MT895862) (bootstrap support 99%) (Fig. 8a). *Amblyomma lepidum* sequences based on the 16S rRNA gene were closely related to *A. lepidum* from Kenya (OQ566211, MT895180, MT895179, OQ566204, and OQ566203) and Somalia (ON532097 and ON532095) (bootstrap support 100%) (Fig. 8b). Analysis of the pairwise distance showed that genetic distance sequences between 12S rRNA and 16S rRNA sequences were minimal (Supplementary file 5).

**Fig. 9:**
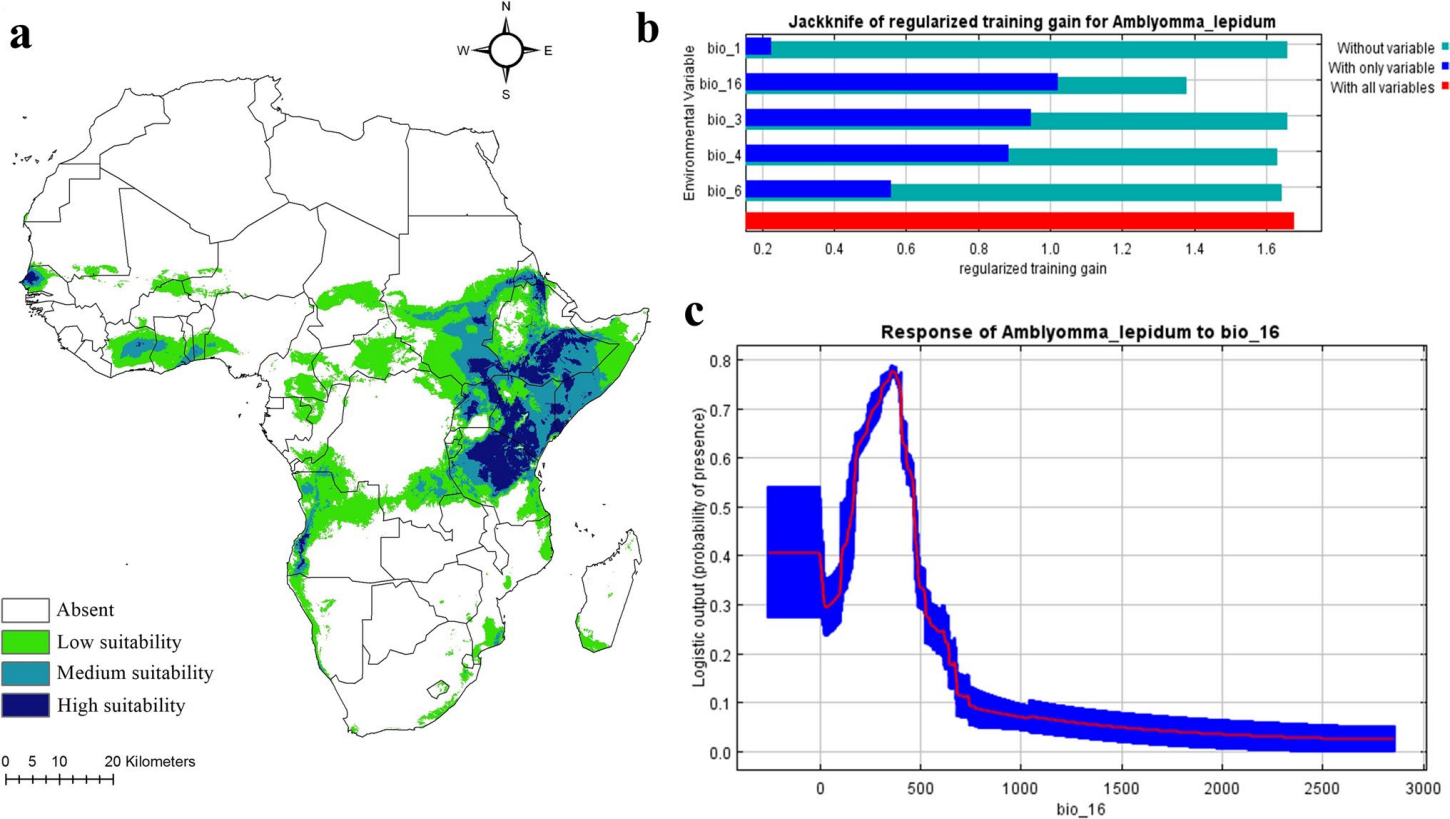
**A** Potential distribution map of *Amblyomma lepidum* in Africa based on climatic conditions during the historical period. **b** The jackknife test showing the most effective environmental variables used in the analysis. **c** Response curve showing the relationships between the probability of the presence of *Amblyomma lepidum* and the top bioclimatic predictor (Bio 16)

**Fig. 10:**
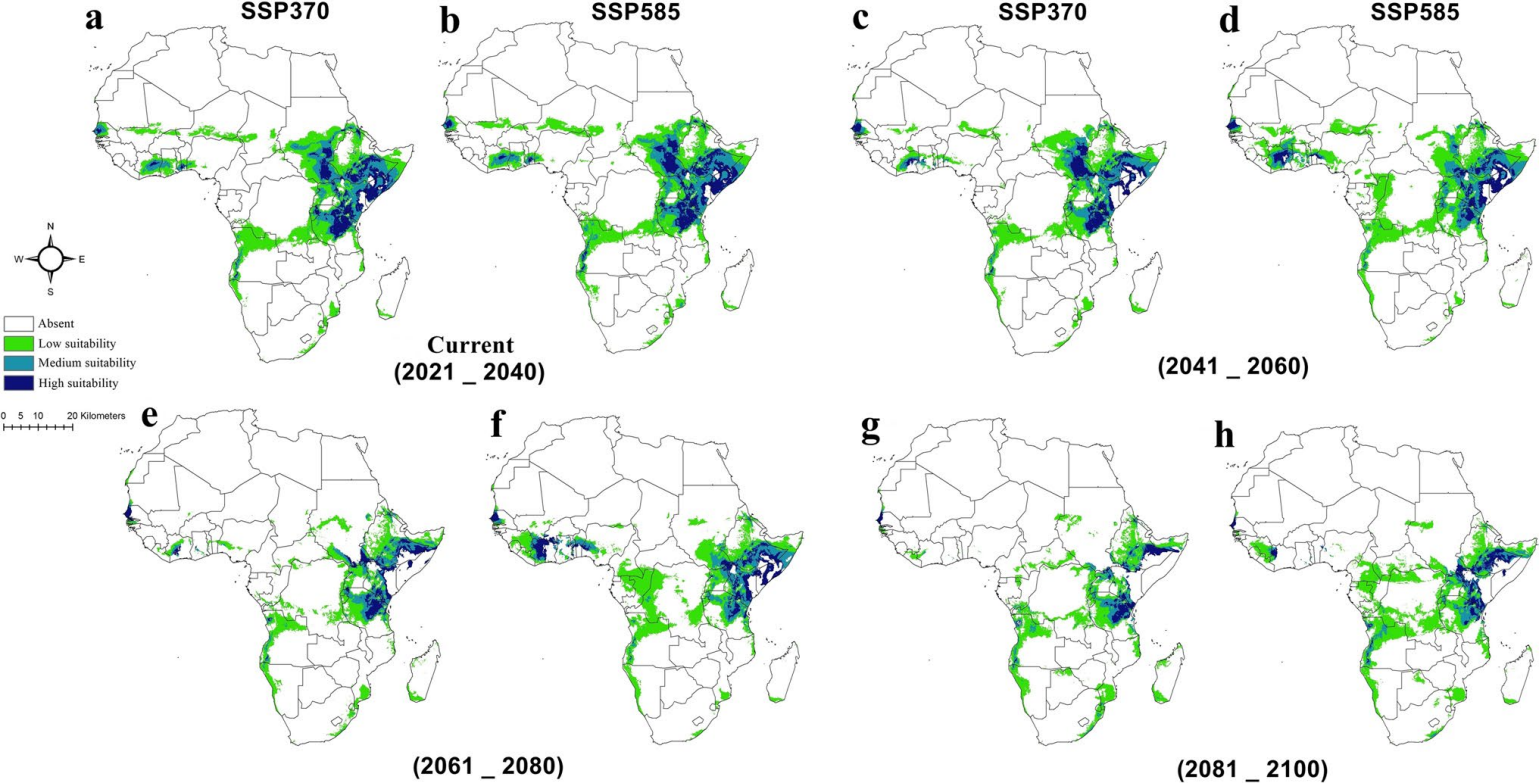
Potential distribution of *Amblyomma lepidum* under different climate change scenarios and Shared Socioeconomic Pathways (SSPs). Maps **a–b** show the potential distribution in 2021–2040, **c–d** in 2041–2060, **e–f** in 2061–2080, and **g–h** in 2081–2100. Predictions are presented for both SSP 370 and SSP 585

Suitable climatic habitats for *A. lepidum* in the historical period (1970–2000) were predicted to be high and medium in Kenya, Ethiopia, Tanzania, Uganda, parts of Northern Eritrea, parts of Southern Somalia, some areas of Southern Sudan, Southwestern Angola, and narrow zones in West Africa, especially in Senegal (Fig. 9a). Lower suitable climatic habitats were predicted in zones of Central and Western Africa (Fig. 9a). Precipitation of the wettest quarter (Bio16) showed higher effects on the potential distribution of *A. lepidum* relative to other predictive bioclimatic variables (Fig. 9b). The climatic habitat suitability of *A. lepidum* decreased sharply with increasing precipitation of the wettest quarter (Bio16) (Fig. 9c). Median of future predictions for two climatic scenarios in SSPs 370 and 585 from 2021 to 2100 showed differences between diverse SSPs from 2021 to 2100 (Fig. 10). For the period from 2021 to 2040, the predictions showed high agreement in suitable climatic habitats compared to the predicted climatic habitats under historical conditions, especially in East Africa. However, the number of highly suitable pixels increased in Southern Somalia in the period from 2021 to 2040, and the number of low suitable pixels in Central Africa decreased in the same period (Fig. 10a, b). For the future climatic conditions influenced by climate change from 2041 to 2100, the suitable climatic habitat is expected to decrease, especially from 2061 to 2100 (Fig. 10c–h).

## Discussion

This study focused on monitoring *Amblyomma* tick species imported from three African countries (Somalia, Ethiopia, and Sudan) to Egypt. All the collected specimens were identified as *A. lepidum* using morphological characteristics and sequence data for the 12S and 16S rRNA genes, which are reliable genetic markers for species identification (Abouelhassan et al. 2019). Although these ticks have been reported in several studies from Egypt (Adham et al. 2009; Hassan et al. 2017; Okely et al. 2021; Abouelhassan et al. 2023), this is the first report of morphological variations and abnormalities for this species. Morphological abnormalities were observed in only one specimen from a total of 57 *A. lepidum* specimens recorded during this study. Though this is the first report of local anomalies in *A. lepidum* from Egypt, a previous study from Uganda (Balinandi et al. 2019) has reported local and general anomalies for the same species. Morphological abnormalities and anomalies in *Hyalomma dromedarii* and *H. rufipes* in ticks from Egypt have previously been observed in the country (Okely et al. 2022a). The observation of anomalous ticks in Egypt in this study and the previous one (Okely et al. 2022a) may be due to antiparasitic treatments used in Egypt. Mutations resulting from antiparasitic treatments such as insecticides and acaricides that hard ticks are exposed to may give rise to morphologically anomalous ticks (Luz et al. 2023).

The distribution of *A. lepidum* is strongly affected by rainfall (Walker et al. 2003), and it prefers arid areas with 250–750 mm of rainfall. The same results were acquired from the response curves (Fig. 9c), indicating this species’ peak distribution in habitats within the range documented in Walker et al. (2003).

The current study also revealed the shape variations of the body shape of male *A. lepidum* using geometric morphometric analysis. Canonical variate analysis (CVA) and principal component analysis (PCA) were employed to assess these variations. CVA effectively separates geographic populations and is sensitive to small statistical trends, while PCA is less sensitive but less prone to bias. The combined results of CVA and PCA indicate statistically significant biological differences among tick populations. The main differences seem to be related to length measurements (body, capitulum, and hypostome), where the larger ticks tend to be in Somalia. Variations in body size among ticks can be attributed to factors such as the quality and quantity of the host’s blood consumed during feeding (Sonenshine 1993; Brunner et al. 2011). Additionally, the habitat of the host may also influence tick size. For instance, tick species that parasitize semi-aquatic animals, like *Amblyomma dubitatum* and *Amblyomma romitii*, exhibit larger spiracular plates (Luz et al. 2020). Environmental effects might also cause morphological differences between different populations of the same species (Hutcheson et al. 1995).

Although the trade of livestock across borders can be financially beneficial, it poses significant health risks. In Africa, the transhumance and trading of livestock can transport ticks carrying pathogens to new regions, potentially leading to the establishment of new tick populations and the spread of tick-borne pathogens (Silatsa et al. 2019; Perveen et al. 2021). For instance, recent studies showed how livestock movement contributes to the geographical expansion of the invasive* Rhipicephalus microplus* tick in new zones in Africa, and subsequently, the pathogens it carries (Madder et al. 2012; Kamani et al. 2017; Ouedraogo et al. 2021; Addo et al. 2023). Cross-border animal trade and unrestricted movements of live animals led to the widespread spread of *R. microplus* in Africa (Silatsa et al. 2019; Kanduma et al. 2020; Muhanguzi et al. 2020). The movement of livestock also plays a pivotal role in the spread of tick-borne diseases (Parola and Raoult 2001). Animal movements were believed to be responsible for the dissemination of diseases such as Rift Valley fever in Africa (Chevalier et al. 2004). Regulations and adopting biosafety strategies governing the movement of livestock serve as an established strategy for controlling infectious diseases with international standards provided by the World Organization for Animal Health (WOAH) (Fèvre et al. 2006). In Egypt, it is crucial to monitor tick species and their associated pathogens on imported wild and domestic animals entering the country (Abouelhassan et al. 2023).

In conclusion, this study provides morphological and molecular analyses for *A. lepidum* ticks collected from imported camels in Egypt. Furthermore, the results suggest that *A. lepidum* has adapted morphology with variations among specimens from the three countries of origin, with minimal potential for genetic divergence. The study highlights the importance of monitoring and controlling the movement of livestock to prevent the introduction and spread of ticks and tick-borne diseases.

### Supplementary Information

Below is the link to the electronic supplementary material.

Supplementary file1 (JPG 369 KB)

Supplementary file2 (DOCX 16 KB)

Supplementary file3 (XLSX 21 KB)

Supplementary file4 (DOCX 19 KB)

Supplementary file5 (XLSX 51 KB)

## Data Availability

All the data generated and analyzed during this study are included in the article. However, raw data is available from the corresponding author upon request. The obtained sequences have been deposited in GenBank (www.ncbi.nlm.nih.gov/genbank/) under accession numbers (OQ947777, OQ947778, OQ947779, OQ947780, OQ947781, OQ947782, OQ947783, OQ947784, OQ947785, OQ955297, OQ955298, OQ955299, OQ955300, OQ955301, OQ955302, OQ955303, OQ955304, and OQ955305).
