# Supplementary figures and images for: Molecular identification and morphological variations of Amblyomma lepidum imported to Egypt, with notes about its potential distribution under climate change
Source: Parasitol Res. 2024 Jul 17;123(7):276. doi: 10.1007/s00436-024-08284-0 (PMC11255089; doi:10.1007/s00436-024-08284-0)

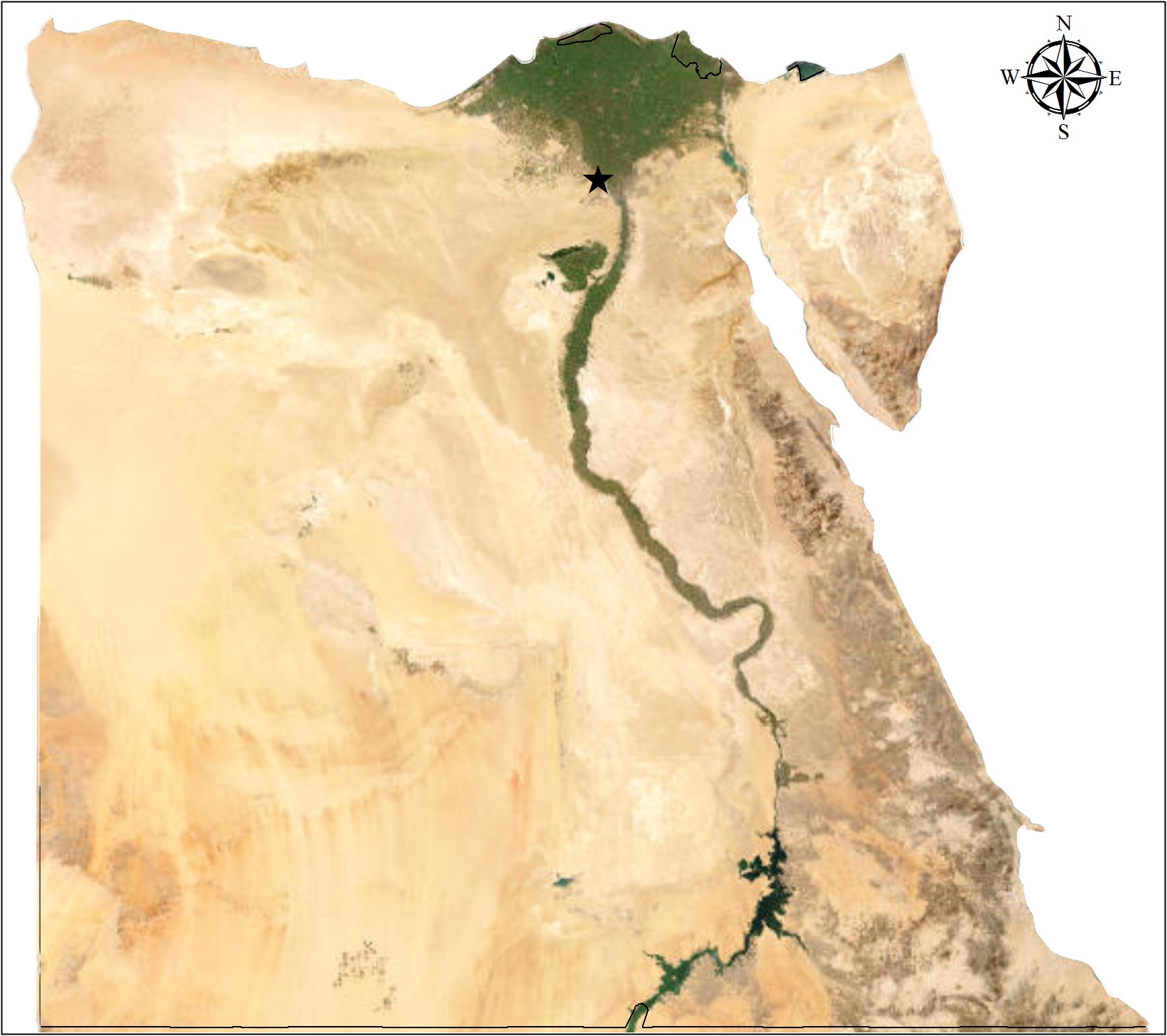

Supplement: Supplementary file 1 — Supplementary file1 (JPG 369 KB) [file 436_2024_8284_MOESM1_ESM.jpg]
