# Supplementary material for: Molecular identification and morphological variations of Amblyomma lepidum imported to Egypt, with notes about its potential distribution under climate change
Source: Parasitol Res. 2024 Jul 17;123(7):276. doi: 10.1007/s00436-024-08284-0 (PMC11255089; doi:10.1007/s00436-024-08284-0)
Supplement: Supplementary file 2 — Supplementary file2 (DOCX 16 KB) [file 436_2024_8284_MOESM2_ESM.docx]

**Supplementary file 2**

Supplementary Table. The initial 19 bioclimatic variables used for the prediction of historical climatic habitat suitability for *Amblyomma lepidum.*

| Variable | Description |
| --- | --- |
| Bio 1 | Annual mean temperature |
| Bio 2 | Mean diurnal range (mean of monthly max temp–min temp) |
| Bio 3 | Isothermality (bio2/bio7) × 100 |
| Bio 4 | Temperature seasonality (standard deviation × 100) |
| Bio 5 | Max temperature of the warmest month |
| Bio 6 | Min temperature of the coldest month |
| Bio 7 | Temperature annual range |
| Bio 8 | Mean temperature of the wettest quarter |
| Bio 9 | Mean temperature of the driest quarter |
| Bio 10 | Mean temperature of the warmest quarter |
| Bio 11 | Mean temperature of the coldest quarter |
| Bio 12 | Annual precipitation |
| Bio 13 | Precipitation of the wettest month |
| Bio 14 | Precipitation of the driest month |
| Bio 15 | Precipitation seasonality (coefficient of variation) |
| Bio 16 | Precipitation of the wettest quarter |
| Bio 17 | Precipitation of the driest quarter |
| Bio 18 | Precipitation of the warmest quarter |
| Bio 19 | Precipitation of the coldest quarter |
