# Supplementary material for: Molecular identification and morphological variations of Amblyomma lepidum imported to Egypt, with notes about its potential distribution under climate change
Source: Parasitol Res. 2024 Jul 17;123(7):276. doi: 10.1007/s00436-024-08284-0 (PMC11255089; doi:10.1007/s00436-024-08284-0)
Supplement: Supplementary file 4 — Supplementary file4 (DOCX 19 KB) [file 436_2024_8284_MOESM4_ESM.docx]

|  | BL | BW | TL | LSI | LSII | LSIII | HL | MAL | LMAL | LMAW | POSL | BCVL | BCVW | BCDL | BCDW |
| --- | --- | --- | --- | --- | --- | --- | --- | --- | --- | --- | --- | --- | --- | --- | --- |
| BL | 1 |  |  |  |  |  |  |  |  |  |  |  |  |  |  |
| BW | -0.0411 | 1 |  |  |  |  |  |  |  |  |  |  |  |  |  |
| TL | 0.38231 | -0.93897 | 1 |  |  |  |  |  |  |  |  |  |  |  |  |
| LSI | 0.9048 | 0.38829 | -0.04758 | 1 |  |  |  |  |  |  |  |  |  |  |  |
| LSII | 0.14839 | -0.99419 | 0.97054 | -0.28686 | 1 |  |  |  |  |  |  |  |  |  |  |
| LSIII | 0.66857 | 0.71554 | -0.43155 | 0.9216 | -0.6362 | 1 |  |  |  |  |  |  |  |  |  |
| HL | -0.95529 | -0.25615 | -0.09201 | -0.99025 | 0.15063 | -0.85855 | 1 |  |  |  |  |  |  |  |  |
| MAL | 0.36727 | 0.91424 | -0.71905 | 0.72838 | -0.86532 | 0.93722 | -0.62585 | 1 |  |  |  |  |  |  |  |
| LMAL | 0.48327 | 0.85487 | -0.62421 | 0.81007 | -0.79406 | 0.97414 | -0.72051 | 0.99178 | 1 |  |  |  |  |  |  |
| LMAW | 0.027885 | 0.99762 | -0.91302 | 0.4509 | -0.98441 | 0.762 | -0.32219 | 0.94 | 0.88861 | 1 |  |  |  |  |  |
| POSL | 0.98651 | 0.12299 | 0.22591 | 0.9623 | -0.01547 | 0.78127 | -0.9908 | 0.51455 | 0.62005 | 0.19112 | 1 |  |  |  |  |
| BCVL | 0.2306 | -0.98171 | 0.98729 | -0.20572 | 0.9965 | -0.56943 | 0.067409 | -0.82036 | -0.74043 | -0.96624 | 0.068222 | 1 |  |  |  |
| BCVW | -0.80346 | 0.62788 | -0.8573 | -0.47344 | -0.70799 | -0.09444 | 0.59151 | 0.25867 | 0.13293 | 0.57272 | -0.69518 | -0.76459 | 1 |  |  |
| BCDL | -0.16879 | -0.97788 | 0.84625 | -0.57245 | 0.94969 | -0.84583 | 0.45267 | -0.97876 | -0.94448 | -0.98998 | -0.32784 | 0.92016 | -0.4512 | 1 |  |
| BCDW | -0.80095 | 0.63115 | -0.85946 | -0.46973 | -0.71096 | -0.09024 | 0.58811 | 0.26274 | 0.1371 | 0.57617 | -0.69214 | -0.7673 | 0.99999 | -0.45495 | 1 |

**Supplementary file4**

Supplementary Table. Correlation coefficient between fifteen morphometric traits in male *Amblyomma lepidum*
